# Thrombosis and Inflammation—A Dynamic Interplay and the Role of Glycosaminoglycans and Activated Protein C

**DOI:** 10.3389/fcvm.2022.866751

**Published:** 2022-03-31

**Authors:** Shrey Kohli, Khurrum Shahzad, Annukka Jouppila, Harry Holthöfer, Berend Isermann, Riitta Lassila

**Affiliations:** ^1^Institute of Laboratory Medicine, Clinical Chemistry and Molecular Diagnostics, University Hospital Leipzig, Leipzig University, Leipzig, Germany; ^2^Clinical Research Institute HUCH, Helsinki, Finland; ^3^Research Program in Systems Oncology, Faculty of Medicine, University of Helsinki, Helsinki, Finland; ^4^Zentrum für Innere Medizin, Universitätsklinikum Hamburg-Eppendorf, Hamburg, Germany; ^5^Coagulation Disorders Unit, Department of Hematology, Comprehensive Cancer Center, Helsinki University Hospital, University of Helsinki, Helsinki, Finland; ^6^Aplagon Ltd., Helsinki, Finland

**Keywords:** thrombo-inflammation, neutrophil extracellular traps (NETs), activated protein C (aPC), glycosamine glycans, platelet-neutrophil complexes, platelet activation

## Abstract

Hemostasis, thrombosis, and inflammation are tightly interconnected processes which may give rise to thrombo-inflammation, involved in infectious and non-infectious acute and chronic diseases, including cardiovascular diseases (CVD). Traditionally, due to its hemostatic role, blood coagulation is isolated from the inflammation, and its critical contribution in the progressing CVD is underrated, until the full occlusion of a critical vessel occurs. Underlying vascular injury exposes extracellular matrix to deposit platelets and inflammatory cells. Platelets being key effector cells, bridge all the three key processes (hemostasis, thrombosis, and inflammation) associated with thrombo-inflammation. Under physiological conditions, platelets remain in an inert state despite the proximity to the endothelium and other cells which are decorated with glycosaminoglycan (GAG)-rich glycocalyx (GAGs). A pathological insult to the endothelium results in an imbalanced blood coagulation system hallmarked by increased thrombin generation due to losses of anticoagulant and cytoprotective mechanisms, i.e., the endothelial GAGs enhancing antithrombin, tissue factor pathway-inhibitor (TFPI) and thrombomodulin-protein C system. Moreover, the loss of GAGs promotes the release of mediators, such as von Willebrand factor (VWF), platelet factor 4 (PF4), and P-selectin, both locally on vascular surfaces and to circulation, further enhancing the adhesion of platelets to the affected sites. Platelet-neutrophil interaction and formation of neutrophil extracellular traps foster thrombo-inflammatory mechanisms exacerbating the cardiovascular disease course. Therefore, therapies which not only target the clotting mechanisms but simultaneously or independently convey potent cytoprotective effects hemming the inflammatory mechanisms are expected to provide clinical benefits. In this regard, we review the cytoprotective protease activated protein C (aPC) and its strong anti-inflammatory effects thereby preventing the ensuing thrombotic complications in CVD. Furthermore, restoring GAG-like vasculo-protection, such as providing heparin-proteoglycan mimetics to improve regulation of platelet and coagulation activity and to suppress of endothelial perturbance and leukocyte-derived pro-inflammatory cytokines, may provide a path to alleviate thrombo-inflammatory disorders in the future. The vascular tissue-modeled heparin proteoglycan mimic, antiplatelet and anticoagulant compound (APAC), dual antiplatelet and anticoagulant, is an injury-targeting and locally acting arterial antithrombotic which downplays collagen- and thrombin-induced and complement-induced activation and protects from organ injury.

## Introduction

Cardiovascular disease (CVD) is the foremost cause of death worldwide, accounting for estimated 17.9 million deaths each year (1). CVDs are a group of disorders that mainly affect heart and blood vessels. CVDs include acute atherothrombotic complications, i.e., myocardial infarction (MI) and ischemic stroke, as well as venous thromboembolic (VTE) disease. Atherosclerosis is the primary underlying disease process driven by lipid accumulation in the arterial wall, persistent inflammation, and vascular endothelial dysfunction (2–4). As it progresses, plaque rupture can occur, to expose blood with the subendothelial matrix. The deliberated plaque content creates an imbalance between the pro- and anticoagulant homeostasis which causes formation of occlusive thrombi (2). Alongside, these thrombotic events not only cause an increased risk for myocardial ischemia and stroke, but they also trigger an interplay between platelets and innate immune cells thereby promoting mechanisms of sterile inflammation. Inflammation and thrombosis are therefore central pathological processes involved in atherosclerosis and associated vascular complications.

Both inflammation and thrombosis in CVD have commonly been investigated independently but been recently integrated within the new concept of vascular thrombo-inflammation (5). In the following sections, we will first outline the thrombotic mechanisms of endothelial damage followed by events leading to activation of inflammatory cardiovascular complications. Finally, we will introduce cytoprotective functions of both conventional and novel therapies, which are known to inhibit to coagulation system, but convey an anti-inflammatory effect independently or aside of blood clotting. One such novel strategy is the provision of heparin proteoglycan-like mimetics, antiplatelet and anticoagilant compound (APAC) (6).

## The Interplay Between Platelets, Coagulation Regulators and Inflammation

### The Interaction Between Platelets and Endothelium

Under physiological conditions, inert platelets circulate unnoticed by other blood cells and vascular endothelium (7). The negatively charged proteoglycans, glycoproteins and glycolipids, together glycocalyx, decorate surfaces of both blood cells and luminal vessel wall and are critical to maintain a quiescent state (8). The protective glycocalyx engages dynamic interaction with its environment, resulting in spatial and organ-specific differences. Majority of the endothelial glycocalyx constituents are protein-bound glycosaminoglycans (GAG), composed of mainly heparan sulfate as well as chondroitin -and dermatan sulfate and non-protein bound hyaluronic acids (9). Endothelial cells carry several mechanisms to regulate and localize the injury, including platelet-inhibitory ectoADPases (CD39), nitric oxide and prostacyclin (10, 11). These mediators not only inhibit platelets, but also tune the vasoactivity and protect from vasoconstriction, local reactions modulating shear rates.

**FIGURE 1:**
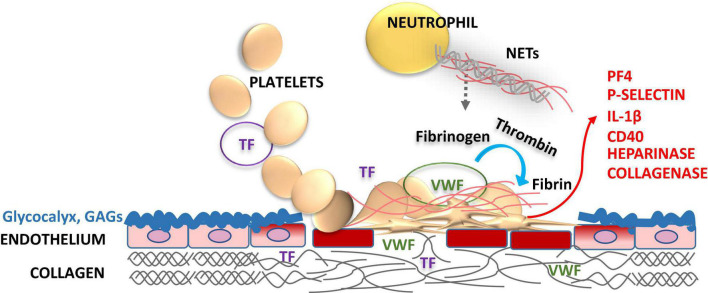
Role of endothelial glycocalyx and platelets in vascular injury. Glycocalyx lines the vascular endothelial cell surface facing the vessel lumen forming a barrier between blood and endothelium maintaining the steady state, but also protects vasculature from pathogens. Glycocalyx supports anticoagulation by enhancing the natural anticoagulants antithrombin (AT), tissue factor pathway inhibitor (TFPI), protein C and S. Disruption of the glycocalyx exposes endothelial cell adhesion molecules [endothelial cell intercellular adhesion molecule-1 (ICAM-1), vascular cell adhesion molecule-1 (VCAM-1), and P-selectin] and extracellular matrix components, i.e., von Willebrand factor (VWF), collagen and tissue factor (TF) in a deeper injury (smooth muscle cells, adventitia). These structures capture blood cells, activating the crosstalk between the inflammatory cells and the coagulation system. Activated platelets release heparinase, metalloproteinases and collagenase to progress the local injury. Activation of platelets feeds back further expression of procoagulant P-selectin, platelet factor 4 (PF4), interleukin-1β (IL-1β), and CD40 ligand to foster the thrombo-inflammatory interactions. Enhanced activation of platelets and leukocytes induces neutrophil extracellular traps (NETs) formation, which in turn activates both inflammatory and hemostatic arms. Vascular endothelial injury and related procoagulant and inflammatory activities may be downplayed by replacing the defective glycocalyx structure with glycosaminoglycan (GAG) moieties, such as APAC.

**FIGURE 2:**
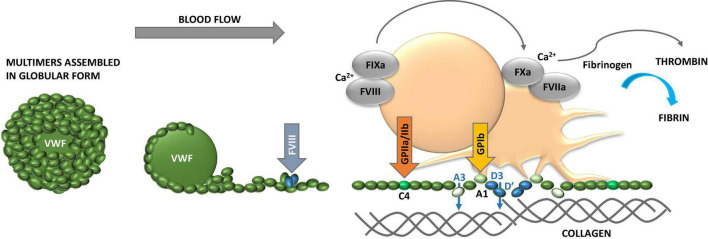
Properties of von Willebrand factor (VWF). VWF obtains globular conformation under static conditions, while it unfolds and elongates under blood flow. The higher the hematocrit, the faster the flow conditions and the smaller the vessel lumen (vasoconstriction), the higher the shear forces and contribution of adhesive platelets. VWF multimer size is controlled by ADAMTS13. While unfolding VWF exposes domains with binding sites for several extracellular proteins, including collagen, and glycosaminoglycans (GAG), and platelet glycoprotein (GP) Ib and GPIIb/IIIa to foster platelet adhesion and activation. VWF carries coagulation factor VIII (FVIII) and liberates it to the tenase complex on platelet surface to support generation of thrombin and formation of fibrin. VWF binds to fibrin as well. The multiple coordinated actions of VWF in platelet recruitment and coagulation pathway are critical in microvascular thrombosis, including thrombo-inflammatory settings.

**FIGURE 3:**
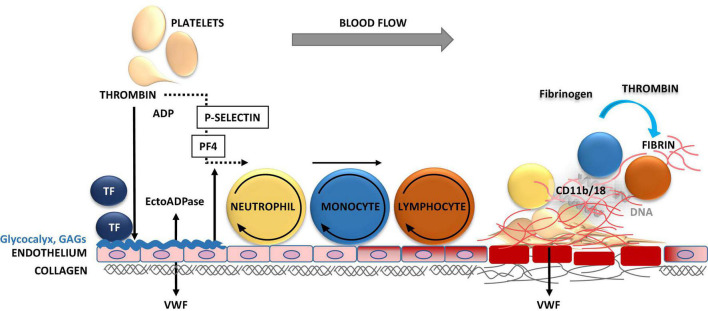
Inflammation-triggered coagulation and thrombosis in vasculature. Upon mechanical injury or systemic inflammation of the vessel wall, activated endothelial cells and monocytes release tissue factor (TF). TF induces the generation of activated forms of coagulation factors FVII, FX, and thrombin, leading to fibrin formation. Thrombin induces the release of P-selectin, which binds to P-selectin glycoprotein ligand-1 (PSGL-1) on neutrophils, monocytes and lymphocytes mediating their tethering and rolling on to the exposed endothelium. The release of procoagulant platelet factor 4 (PF4) and von Willebrand factor (VWF) promote the recruitment, adhesion, and activation of platelets and leukocytes at the injury site. Thrombin cleaves fibrinogen to form fibrin, which stabilizes thrombosis. The firm adhesion of leukocytes to fibrin occurs via CD11b/18 αMβ2, macrophage-1 antigen (MAC-1) or complement receptor 3 (CR3).

Platelets are the first blood cells to respond to vascular injury, regardless of the cause of damage; intervention, or disease state (12, 13). Injury causes the loss, shedding or alteration of the balanced glycocalyx structure and, thus, exposure of underlying adhesive proteins to interact with blood (Figure 1) (14–16). Platelets adhere to injury-exposed subendothelial matrix components, where selected binding depends on the shear rate of the flowing blood (7). Under arterial and microvascular shear rates platelets are first arrested from the blood flow by interaction of glycoprotein receptor (GP) Ib-V-IX and von Willebrand factor (VWF) and tightened by GPIIb/IIIa, engaging, also, fibrinogen and fibrin while the blood flow is altered by the growing thrombi (Figures 1, 2). Also, inflammatory cells roll and tether on the inflamed endothelium and bind to fibrin via their αMβ2 (CD11b/18) integrin receptors (Figure 3) (17).

Permanent platelet adhesion is secured by the early binding to matrix proteins, mainly collagen, fibronectin, and laminin via GPIa/IIa, GPVI and integrin α6β1 receptors, respectively (18, 19). In turn, under venous shear rates, platelets may bind directly to the forming fibrin, once the natural anticoagulation, provided by antithrombin, thrombomodulin – protein C and protein S as well as tissue factor pathway inhibitor (TFPI), starts to fail. The subsequent activation of platelets is enhanced by thrombin, and platelet-and red cell-released adenosine diphosphate (ADP), and enzymatic generation of thromboxane A_2_ from arachidonic acid (18, 20–24). Recruitment of coagulation factors to foster thrombin formation, platelet aggregation and contraction, as well as fibrin mesh are all stabilizing the platelet plug at the vascular injury site. In addition to their role in coagulation, versatile platelets contribute widely to inflammation and immunity (12, 25, 26). Inflammation-associated endothelial damage underlies several cardiovascular, hematological, and kidney complications: ischemia reperfusion injuries (IRI), sepsis, thrombo-inflammation, and microangiopathy causing organ hypoxia. In response to hypoxia, endothelial cells will release further adhesive components, i.e., VWF/FVIII to the vascular surface and circulation (27) maintaining pro-inflammatory and thrombotic conditions (Figures 1, 2). Damage to endothelium is associated with shedding of heparan sulfate from glycocalyx due to heparinases, which are released by activated platelets and provided by several bacteria.

Thus, functional endothelium and its glycocalyx and other regulatory capacities keep the platelet, coagulation and inflammatory response localized and finely balanced to prevent thrombosis from spreading.

Damage to the endothelium results in release of mediators both locally on vascular surfaces and to circulation, further enhancing platelet adhesion to the affected sites, especially in microcirculation (Figures 1, 2). These key mediators being both platelet- and endothelium-derived, include VWF, Platelet Factor 4 (PF4, CXCL4) and P-selectin (28). These mediators interact with neutrophils at the injury site to arrest them from blood flow to clear up the inflammatory plaque (Figure 3). In addition to its role in platelet adhesion and aggregation, and fibrin binding, VWF also mediates extravasation of leukocytes, an important element of inflammation with tissue injury (29). In this section, we will outline the contributions of VWF, PF4 and P-selectin, for thrombo-inflammatory pathways.

#### Von Willebrand Factor

Von Willebrand factor obtains globular conformation under static conditions to unfold under active blood flow (Figure 2) (30). This is relevant to the bench studies which overlook blood flow. The largest multimers are released from activated platelets, and in case the size regulating ADAMTS13 enzyme is deficient, such as occurs in thrombotic thrombocytopenic purpura (TTP) (31). Sticky VWF binds to several extracellular proteins, including collagen, and GAG structures and to platelet GP Ib and IIb/IIIa to foster platelet adhesion and activation and to resist blood flow forces. This VWF-platelet interaction is highly relevant under high blood flow conditions, including microcirculation and stenosed larger arteries. The larger the multimers, the higher the hematocrit, the faster the flow conditions and the smaller the vessel diameter, the higher are the physical shear rates and the more platelets deposit on VWF (19).

Von Willebrand factor also carries coagulation factor FVIII to platelet surfaces to support generation of thrombin and formation of fibrin, where VWF also binds to. The multiple coordinated actions of VWF in platelet recruitment and coagulation pathway are critical in microvascular thrombosis, including thrombo-inflammatory settings (32). Due to neutrophil-induced oxidation VWF cleavage by ADAMTS13 is impaired, creating large multimers which deposit platelets on micro-vasculature and cause organ damage and thrombocytopenia (Figure 1). VWF is also involved in mediating extravasation of leukocytes (33). One example of infection is adenovirus-induced endothelial activation which leads to the above mediator release and platelet activation, and subsequent binding of virus to platelets causes their clearance and thrombocytopenia (34).

#### Platelet Factor 4

Platelet factor 4 is the early platelet-released chemoattractant for neutrophils, but also for monocytes and fibroblasts (35). Upon activation, platelets release procoagulant PF4, which neutralizes negatively charged heparin-like GAGs and their anticoagulant potential, including the glycocalyx, and the therapeutic unfractionated heparin (UFH), but less so the lower molecular weight species (LMWH, low-molecular weight heparin).

As a chemokine, PF4 has a well-established role in inflammation, but it also poses an immunological epitope together with polyanionic structures, including DNA or GAGs (36). PF4-polyanion interactions trigger formation of antibodies of IgG4 class to induce prothrombotic heparin-induced thrombocytopenia (HIT), and recently discovered adenoviral vectored vaccine-induced thrombocytopenic and severe thrombotic syndrome (VITT), without exogenous heparin exposure (37).

Platelet factor 4 together with P-selectin provides a signal for leukocyte rolling on endothelial surfaces and serves as the early ligand for platelet-neutrophil interactions (Figure 3). Moreover, PF4 triggers vascular smooth muscle cell proliferation, which upon regulation is involved in vascular healing, however, when escaping from its regulation by, e.g., GAGs, PF4 will turn into a harmful player inducing proliferation of arterial wall, introduction of pulmonary hypertension, as one devastating example (38).

In this regard, therapeutic heparin has several anti-inflammatory properties, and when administered early to patients with infection, i.e., SARS-CoV-2 lately at treatment doses, even without visible venous thromboembolism, heparin is lifesaving. Heparin been shown to reduce not only early PF4-related activities, but also P-selectin-mediated leukocyte recruitment under blood flow, and further even cancer metastasis has been shown to be attenuated by a background of P-selectin deficiency or by treatment with heparin (39).

#### P-Selectin

**FIGURE 4:**
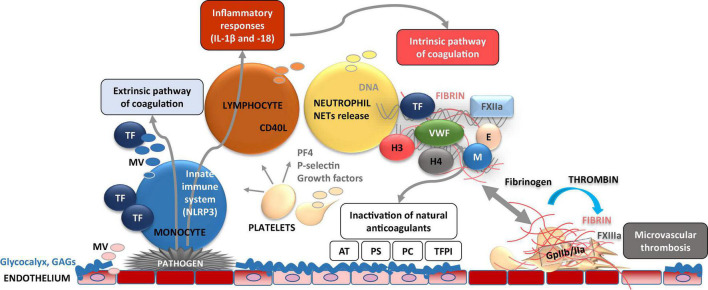
Dynamic cellular and molecular interplay underlying thrombo-inflammation. Pathogen- or danger-signal mediated activation of endothelial cells at the site of disrupted glycocalyx results in adhesion of immune cells (e.g., monocytes) on the cell surface. Activated endothelial and immune cells release microvesicles (MV) bearing, e.g., tissue factor (TF) which induces activation of extrinsic coagulation pathway. TF is also capable to activate NLRP3 inflammasome [subsequent activation of interleukin (IL)-1β, and IL-18] and inflammatory response. Furthermore, endothelial cell activation results in adhesion of platelets which act as chemo-attractants (e.g., via P-selectin) to neutrophils. As consequence, neutrophil activation results in formation and release of neutrophil extracellular traps (NETs) composed of several pro-inflammatory molecules, neutrophile elastase (E), myeloperoxidase (M), histones (H3 and 4), which perpetuate endothelial dysfunction. NETs activate coagulation FXII and the intrinsic pathway of coagulation. Subsequent enhanced fibrin formation leads to the capture and kill of pathogens in fibrin-strengthened NETs. Procoagulant platelets also release platelet factor 4 (PF4) which inactivates glycosaminoglycans (GAGs) such as heparin. On the other hand, these thrombo-inflammatory mechanisms are accompanied by inactivation of cytoprotective anticoagulants such as activated protein C, antithrombin (AT), tissue factor pathway inhibitor (TFPI), and protein S.

CD62P, P-Selectin is a thrombo-inflammatory molecule which is exposed on the platelet and endothelial surface upon activation (Figures 3, 4). It is therefore a key molecule that further promotes activation and aggregation of platelets. It is not solely an adhesive molecule, its binding to the P-selectin glycoprotein ligand-1 (PSGL-1) induces platelet activation and enhances aggregation (40). P-selectin blocking antibodies are therefore helpful in preventing both venous thrombosis and vessel wall inflammation (41). In addition, also heparan sulfate, heparin proteoglycans and UFH inhibit P- and L-selectin binding with ability to interfere with PSGL-1 (42). Importantly, the receptor PSGL-1 is widely expressed by leukocytes. Upon thrombo-inflammation, platelets can therefore influence leukocyte migration and activation. Interestingly, neutrophils abundantly express PSGL-1 and search for P-selectin expressing platelets and to coordinate thrombo-inflammation. Such platelet-neutrophil complexes promote the expression of endothelial intercellular adhesion molecule 1 (ICAM-1), which is necessary for neutrophil extravasation into the organ (43). These processes have been explained in several organ and disease systems, i.e., pulmonary infection, renal IRI or bacterial infection in the liver (44–46). An interaction, which was initially thought to promote phagocytic clearance of bacteria, is increasingly known as formation of neutrophil extracellular traps (NETs), which will be discussed next.

## Neutrophil Extracellular Traps at the Cross-Roads of Thrombo-Inflammation in Cardiovascular Diseases

An interaction of platelets with immune cells promotes the onset of inflammatory processes (Figures 3, 4). In infectious diseases, neutrophils upon activation result in formation of neutrophil extracellular traps (NETs) to sequester or kill the pathogens (13). As outlined in the previous section, platelet activation and surface expression of P-selectin and secretion of PF4 enable platelets to bind to neutrophils, which result in their activation and further recruitment to sites of tissue damage. This will initiate the formation of neutrophil extracellular traps (NETs).

However, NET formation is not limited to infectious diseases but plays a role in both acute as well as chronic sterile inflammatory diseases such as atherosclerosis, diabetes and kidney diseases (47). An exacerbated release of NETs has both pro-thrombotic and pro-inflammatory effects and induces endothelial dysfunction. Platelets are a part of NETs and are major contributors to acute innate inflammation. Platelets express pattern recognition receptors, resulting in their activation upon contact with danger-associated molecule patterns. Interactions between neutrophils and platelets trigger and accelerate NET formation, as well as thrombosis due to aggregation of platelets. In addition, abnormal activation of neutrophils may lead to endothelial damage during autoimmune or exaggerated inflammatory responses by releasing neutrophil serine proteases into the circulation, which activate specific cell surface receptors (48, 49).

Neutrophil extracellular traps are present within human and mouse atherosclerotic lesions (50). Their presence next to the apoptotic endothelial and smooth muscle cells within the plaques imply that they contribute to plaque disruption (51). Another study showed that neutrophils and NETs localized in all types of complicated lesions, without differences between ruptures, erosions, and intraplaque hemorrhages (52). On the other hand, NETs were not present within intact plaques, but they were numerous within adjacent perivascular tissue of complicated plaques. Despite an association and experimental evidence for the involvement of NETs in atherothrombosis, the mechanisms whereby they are either a cause or a consequence of plaque instability remain to be shown (52).

In further studies, mitochondrial oxidative stress has been associated with NETs and lesion size. Thus, a causal link was identified between endogenous neutrophil mitochondrial oxidative stress level with NETosis and atherosclerotic lesions in aged mice (53). However, these studies do not identify an association with thrombo-inflammatory mechanisms and NET formation. On the other hand, several studies show that NETs are capable of aggravating thrombotic complications of atherosclerotic plaques, including plaque disruption (54). Furthermore, plasma levels of myeloperoxidase (MPO) and MPO-DNA complex correlate with a risk of coronary artery disease and other major adverse cardiac events suggesting that NETs, and associated biomarkers can be used to predict a risk for atherosclerotic disease burden and events (54, 55).

Neutrophil extracellular traps not only promote thrombin generation, but also possess prothrombotic molecules which include tissue factor (TF), FXII, histones H3 and H4, and fibrin(ogen) (56). Therefore, they play a major role in arterial as well as venous thrombosis. An interaction of neutrophils and platelets at the site of plaque rupture promotes NET formation, thereby increasing TF abundance and prothrombotic events. Neutrophil-mediated platelet aggregation via integrins (α9β1) promotes arterial thrombosis. Vice versa, platelets promote NETosis. As outlined above, P-selectin is an important mediator which along with other adhesion molecules mutually mediates the interaction between platelets and neutrophils. Accordingly, beyond the beneficial actions of routine heparin use, P-selectin blocking antibody inclacumab has been proven to be beneficial in non-STEMI patients in preventing myocardial damage. Similarly, inhibition of NETs by PAD4 deletion has been shown to abrogate NET associated atherosclerosis burden and inflammatory response (57). Furthermore, PAD4 dependent NETosis is associated with plaque rupture and erosion (51). These observations suggest that NETs participate in thrombotic complications of atherosclerosis. Furthermore, studies in mouse models show that NETs impair endothelial cell survival under such conditions (58). Taken together, an interplay between neutrophils, platelets, and hemostatic factors are important mediator of the pathophysiology of cardiovascular diseases (Figures 3, 4).

Simultaneously with promoting activation of the hemostatic system, NETs also contribute to activation of inflammatory pathways enhancing the atherothrombotic processes. NET-mediated activation of the inflammasome can amplify the inflammatory response through a feed-forward loop. The inflammasome stimulation triggers synthesis and release of interleukin (IL)-18 and IL-1β, which in turn enhance NET formation (59). NLRP3 inflammasome-associated activation of IL-1β and IL-18 is identified as an essential pathogenic mechanism in CVD and its inhibition reduces IL-6 synthesis (60, 61), in addition to that of fibrinogen. Also, the association with inflammasome, neutrophils possess armaments which include reactive oxygen species (ROS), lipid mediators (e.g., eicosanoids) as well as granular proteins such as alarmins (e.g., cathelicidins, defensins), MPO and serine proteases. These enzymes bind to NETs and promote/support local inflammatory functions at the site of NET release. Mechanistic studies on how these granular proteins, when conjugated with NETs, modify organ function are not yet reported.

### Neutrophil Extracellular Traps and Glycosaminoglycans

Glycosaminoglycans and sialylated glycans play an important role in cellular signaling and immunological events. Regarding neutrophils and NETs, it has been observed that glycophorin A, a glycoprotein on erythrocyte, acts as a natural inhibitor of neutrophil activation in circulation (62). Thus, neutrophils are more susceptible to undergo NETosis under isolated cell culture conditions. Besides sialoglycoproteins, and neutrophil activation, NET formation is largely dependent on heparin sulfate. In this regard, heparin, a potent anticoagulant, is known to inhibit neutrophil elastase activity (63). In this study, sulfation was required for inhibition of neutrophil aggregation and elastase activity, since a non-sulfated GAG, hyaluronic acid and neutral dextran, were unable to support the elastase -induced inhibition of neutrophils (63).

Besides inhibiting elastase activity, heparin is able to destabilize histones and destroy the NET scaffold, thereby preventing thrombus formation (64, 65). Endothelial GAGs support the chemotaxis of neutrophils (66). Furthermore, GAGs on the surface of neutrophils are known to synergistically act with GAGs on the inflamed tissues mediating their migration. This indicates that GAGs or GAG-mimetics are a promising therapeutic approach to prevent neutrophil-mediated thrombo-inflammatory effects. However, a direct evidence of GAG-dependent neutrophil activation and NETs in CVD remains to be evaluated.

## Activated Protein C and Thrombo-Inflammation in Cardiovascular Diseases

**FIGURE 5:**
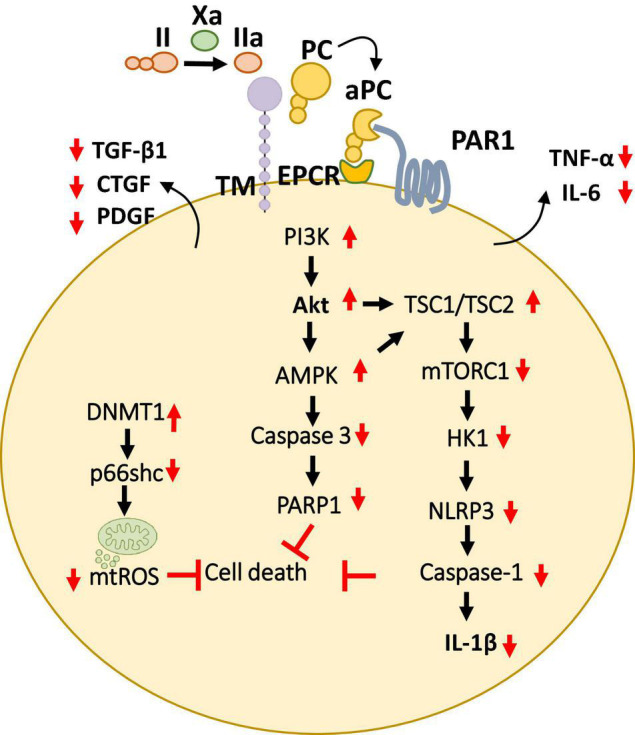
Overview of cytoprotective signaling mechanisms in the myocardium. aPC triggers a complex signaling network of PI3K thereby inhibiting cell death and promote cardioprotection by preventing the mitochondrial ROS and pro-apoptotic as well as inflammasome associated caspases described in multiple cell types of the myocardium. Additionally, aPC has been shown to prevent the release of pro-inflammatory cytokines (TNF-α, IL-6) as well as pro-fibrotic factors (TGF-β, CTGF, PDGF) from the myocardium. Bottom pointing red arrows, downregulation; Upward pointing red arrows, upregulation; Black arrows, pathway connectors.

In addition to procoagulant function, thrombin is also an integral component of thrombomodulin, protein C (TM-PC) system. Protein C is serine protease zymogen that is synthesized in the liver, circulates in the plasma and has a high affinity to endothelial protein C receptor (EPCR). Activation of protein C (aPC) is mediated by the thrombin and TM (thrombomodulin) complex on the endothelial surface and is, therefore, thrombin-dependent at its moderate concentrations (67). aPC is well known for its anticoagulant and cytoprotective functions (68–70). The anti-coagulant activity of aPC is dependent on its ability to inactivate FVa and FVIIIa, thereby inhibiting thrombin generation (71–73). Furthermore, aPC confers cytoprotective signaling largely via protease activated receptor 1 (PAR1) on endothelial cells (Figure 5). Interestingly, other PAR receptors in combination of cellular receptors (e.g., integrins) have also been shown to contribute to cytoprotective mechanisms of aPC.

Plasma aPC levels decline and are inversely linked with clinical severity of coronary artery atherosclerosis and with cardiac ischemic injury in patients (74). Likewise, TM and the endothelial cell protein C receptor are downregulated on endothelial cells overlying the atherosclerotic plaque in coronary arteries (75). Several preclinical studies have shown cardio-protective effects of aPC (76–79). aPC restricts cardiomyocyte cell death in myocardial IRI, a hallmark of thrombo-inflammation (77, 78).

These protective effects were dependent on PAR-1 and were paralleled by an anti-inflammatory effect based on lowered IL-6 levels and leukocyte infiltration (78) (Figure 5). Furthermore, to mediate these anti-inflammatory, cardioprotective effects in cardiac IRI, independent of its anticoagulant activities aPC stimulates AMPK signaling but inhibits NF-κB and JNK signaling (79). The generation of signaling competent aPC variants (e.g., 3K3A, PC-2Cys) which do not interfere with the hemostatic activities has enabled to strengthen these cytoprotective effects of aPC in CVD (79–81). Moreover, the non-anticoagulant variants 3K3A-aPC and PC-2Cys, but not the non-signaling aPC-E170A mutant, restricted induction of pro-inflammatory TNF-α and IL-6 following myocardial IRI (79, 81). Interestingly, aPC conveys autophagic activities through AMPK driven metabolic changes thereby mediating cardio-protection in IRI (82, 83). Congruent to these observations, aPC administration limited myocardial IRI-triggered NLRP3 inflammasome activation via an AMPK -driven mechanism involving restricting of rapamycin kinase complex 1 (TORC1) signaling and hexokinase 1 (Figure 5). The protective effect of aPC was mimicked by 3K3A-aPC or parmodulin-2, a biased PAR-1 modulator (81).

Compatibly, aPC was shown to limit hypoxia reoxygenation (H/R)-induced NLRP3 inflammasome activation in neonatal murine cardiomyocytes and cardio-fibroblasts, the vital cells of the heart (81). These studies established that aPC is an endogenous negative regulator of NLRP3 inflammasome activation following IRI, uncovering a new anti-inflammatory mechanism of aPC. In our recent follow-up studies, we have investigated the transcriptomic profile of aPC treatment in murine myocardial IRI in comparison to direct oral anticoagulants FXa inhibitor (FXai, rivaroxaban) and FIIa inhibitor (FIIai, dabigatran). In this regard, the dosing regimens for both anticoagulants were experimentally determined to provide comparable anticoagulant effects and the infarct sizes (84). The results from our study show that the gene expression profile of aPC-treated mice resembled that of mice treated with FXa inhibitor (FXai, rivaroxaban) (84). On the contrary, mice treated with FIIa inhibitor (FIIai, dabigatran) had a markedly different gene expression profile compared to FXai or aPC treated mice. Alike aPC, FXai prevented the NLRP3 inflammasome activation following IRI. These protective anti-inflammatory effects of FXai, depended on aPC generation and were lost following endogenous aPC blockade. While both FXai and FIIai are potent anticoagulants and used to prevent cardiovascular complications, the observed gene expression changes in our study were independent of their anticoagulation efficacy.

Activated protein C-mediated cardio-protection is not limited to myocardial IRI model. Since AMPK is known to play a protective role in pressure-overload induced cardiac hypertrophy, aPC was found to confer cardio-protection in mouse models (85). Interestingly, in this model, aPC was able to prevent macrophage infiltration and the activity of redox enzyme p66*^shc^*, thereby inhibiting ROS accumulation. Moreover, we have recently demonstrated that aPC reduces epigenetically sustained redox regulator p66*^Shc^* to avert diabetes-induced accelerated atherosclerosis (86). Mechanistically, in this study aPC mediated reversal of glucose-induced CpG hypo-methylation within the p66*^Shc^* promoter by induction of the DNA methyltransferase-1 (DNMT1) was demonstrated as the critical signaling axis in the athero-protection (86) (Figure 5). aPC also ameliorated angiotensin II- triggered myocardial remodeling by limiting expression of the pro-fibrotic cytokines transforming growth factor beta 1 (TGF-β1), connective tissue growth factor (CTGF), and platelet-derived growth factor (PDGF) (87) (Figure 5). In this context, aPC was found to confer cardio-protection by acting on the infiltrating immune cells. Overall, these studies suggest a general anti-inflammatory, cytoprotective role of aPC in mediating cardio-protection independent of its role in blood clotting.

Taken together, these studies highlight a coagulation-independent role of aPC in preventing inflammatory mechanisms in CVD. The current studies have focused on the role of aPC in cellular signaling in specific cell types. As a result of these cytoprotective mechanisms, the overall cardiovascular health is restored thereby avoiding further thrombotic complications.

### Activated Protein C and Glycosaminoglycans

Proteoglycans have been long known to regulate thrombin. Chondroitin moieties are important for the binding of thrombin and thrombomodulin, which is necessary to produce aPC. Heparin and chondroitin sulfate interact with arginine residues on thrombin and regulate its activity, thereby also controlling protein C activation (88, 89). Furthermore, serpins which are predominant protease inhibitors, capable of effectively inhibiting aPC, are in turn regulated by interactions with GAGs, such as heparin or heparan-sulfate (90). Interestingly, an interaction of FXa with anionic phospholipids, influences its binding to GAGs, thereby allosterically modulating the active site of FXa, and enhances its capacity to activate protein C (91). These findings suggest that modulating the GAG function can indirectly modulate protein C activation, to regulate thrombosis as well as anti-inflammatory cellular mechanisms. Therefore, therapies which can mediate these effects, could act as a double-edge sword against cardiovascular complications.

## Heparin Proteoglycan-Mimetic, Antiplatelet and Anticoagilant Compound

Antiplatelet and anticoagulant compound APAC is a heparin proteoglycan mimetic, in which UFH chains are covalently conjugated to globular protein core of albumin. The conjugation reaction can be modified to provide highly negatively charged concentrated GAG moieties with tailored number of heparin chains with functional impacts (6, 92). APAC has the dual properties of inhibiting both collagen- and thrombin-induced platelet activation and aggregation and act as an anticoagulant, by virtue of the heparin moieties (6). Intravenously injected APAC dose-dependently prolonged activated partial thromboplastin time (APTT) in plasma without any accumulation effect during 2-week daily repeated dosing in rodents and non-human primates (93). APAC is more potent anticoagulant than UFH when measured with thrombin time in human and animal plasma. APAC followed similar clearance route to UFH, mainly via liver and kidneys (94).

In a severe model of local thrombosis on TF and collagen surface, human blood collected to APAC alone (without any other anticoagulation) and subjected to high shear blood flow, lead to reduced deposition of both platelets and fibrin (92). In a baboon Folts model of arterial thrombosis upon severe stenosis (30–90%) APAC maintained patency of the artery when administered locally (6). As such, APAC acts as a dual platelet and thrombin inhibitor, to control VWF-mediated thrombus growth under high shear force-blood flow conditions.

Direct binding of purified VWF to APAC was shown by immunoprecipitation analysis (92). In addition, based on atomic force microscopy studies, recombinant VWF supplemented to APAC solution reduced APAC binding on muscovite mica surface, supporting competitive interaction of VWF to APAC (95). APAC can also interact with platelet- and megakaryocyte-specific receptor G6b-B, the immunoreceptor tyrosine-based inhibition motif (ITIM)-containing receptor, which is critical for platelet production and activation (96). The effect of APAC and UFH, on wild type and G6b-B deficient mouse platelets was studied using flow cytometry and by detecting GPIIb/IIIa activation-induced fibrinogen binding and by platelet degranulation (TLT-1 expression) with or without stimulation of C-type lectin like receptor 2, CLEC-2 antibody (96–98). CLEC-2 triggers the downstream semi-immunoreceptor tyrosine-based activation motif (ITAM) pathway, and dimerization of CLEC-2 leads to binding of Spleen Tyrosine Kinase (Syk) and subsequent further progression of downstream tyrosine phosphorylation events and eventually platelet aggregation (97). The multiple functions of CLEC-2 have been discussed in several reviews including addressing its role to control collagen-induced platelet activation and thrombo-inflammation (99–102). APAC, unlike UFH, inhibited CLEC-2 receptor stimulation-induced platelet activation and degranulation in wild-type platelets, but not with G6b-B deficient platelets. Thus, APAC may suppress CLEC-2 mediated platelet activation by inducing an inhibitory signal via G6b-B (96). Using platelets from humans and genetically modified mice, interaction of G6b-B to heparins, and more so to APAC, inhibited platelet and megakaryocyte functions (96). Toxicology studies did not show thrombocytopenia (93).

Upon APAC exposure, the injury-induced inflammatory complications can be avoided or decreased in the ruptured vascular endothelium model. An intriguing suggestion is to limit subsequent damages by regeneration of the glycocalyx with an injury site- targeting glycoprotein structures, such as APAC, to speed up the healing process (103). In animal models, both locally and systemically administered APAC was, indeed, shown to target to the surgically crafted injury under normal and high flow conditions in an acute setting (6, 103). Notably, APAC but not UFH, rescued kidney function, and inflammation alleviated in an IRI rat model of acute kidney injury (94). Also, inhibition of complement (anti-Complement compound 5 antibody, BB5.1) reduces glycocalyx shedding and IRI damage in mice model of acute kidney injury (104).

**FIGURE 6:**
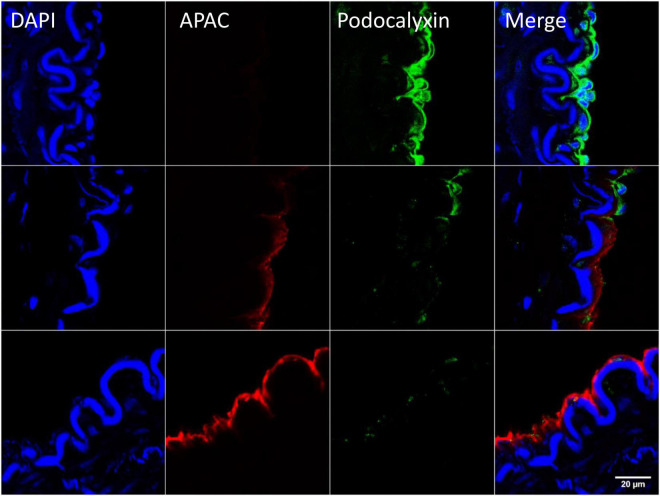
Example of targeting and binding of the heparin proteoglycan mimetic, APAC at the injured arterial wall *in vivo*. APAC and podocalyxin double staining on the arterial injury of vascular anastomosis in pig (103). The anastomotic area was treated with biotinylated APAC (0.3 mg) before exposing to circulation. After 30 min of restored blood flow the artery was resected and processed for histology. Histological samples were stained for nuclei with 4′,6-diamidino-2-phenylindole (DAPI) (blue), APAC (red), and podocalyxin (green). Podocalyxin is a glycosylated cell surface sialomucin expressed, e.g., by vascular endothelial cells and hematopoietic progenitors (107). Disrupted endothelium is depicted by the binding of APAC and the intact endothelium by the binding of podocalyxin. APAC signal was absent at the sites of podocalyxin (merge). Scale bar corresponds to 20 μm.

After exposure of internal elastic lamina during severe balloon injury, immunohistochemical analysis of the biotin-labeled APAC confirmed the co-localization of highly negatively charged APAC with positively charged VWF (103). In contrast, at the site of preserved endothelium, i.e., when platelet endothelial cell adhesion molecule (PECAM-1, CD31) (105) or podocalyxin (106) was present, APAC was absent (103) (Figure 6). The similar colocalization of APAC with VWF and laminin was depicted at anastomosis sites of arteriovenous fistula (AVF) surgery (103). In AVF, very high shear rates prevail, and maintenance of the conduit is jeopardized by a high risk of closure already prior its uptake for clinical use (maturation failure), and when used for hemodialysis access after repeated (thrice a week) exposure of blood to collagen with dialysis needle punctures. These indications provide a clinical unmet need, which APAC treatment may address.

Moreover, data are evolving that APAC also targets the injury site from blood circulation in two mouse models of vascular surgery and laser injury, both preventing and delaying thrombus formation. In the reversible kidney IRI model, APAC reduced expression of innate immunity ligand, hyaluronan, tubule-interstitial injury marker, Kim-1, and alleviated structural damage of the renal cortex (94). Specifically, neutrophil gelatinase-associated lipocalin, a marker of renal epithelial injury was reduced, while serum creatinine and urea nitrogen showed a timely fall. Furthermore, in this irreversible IRI model, APAC reversed the kidney failure and reduced serum levels of vascular destabilizing and pro-inflammatory angiopoietin-2 and syndecan-1. UFH was unable to provide this array of protection (94). In conclusion, reno-protection effect of APAC was evident following both a reversible IRI and even after a severe, irreversible IRI by attenuating vascular injury and innate immune activation.

Given its association to GAGs, APAC not only mediates antiplatelet and anticoagulant properties but can confer additional cytoprotection by promoting the activation of protein C. However, it remains to be shown if APAC treatment will enhance of aPC at the site of inflammation or in circulating blood.

## Conclusion

The hemostatic and the inflammatory systems are inter-connected. Coagulation regulators, classically known to solely contribute to the hemostasis machinery and mediate blood clotting have been increasingly recognized to regulate cellular processes including inflammation. Activated platelets are the key players during thrombosis, and in turn trigger inflammation and immune responses, either directly or via activation of immune cells. In this regard, neutrophil activation, and formation of NETs importantly contribute to the pathophysiology of cardiovascular disease by linking thrombosis and inflammation, popularly termed as thrombo-inflammation. Likewise, activation of inflammatory response is paralleled by altered regulation of coagulation proteases and platelet activation. Therefore, antithrombotic therapies which have a dual mode of action, and not only prevent clotting but can confer cytoprotective effects are beneficial in resolving thrombo-inflammation. One such strategy could be heparin proteoglycan mimetic, which locally targets several above key protective functions at sites of vascular damage.

## Author Contributions

SK, KS, AJ, HH, BI, and RL contributed to the interpretation of the available data and writing of the manuscript. All authors approved the manuscript.

## Conflict of Interest

RL is the CSO for Aplagon Ltd., Helsinki, Finland. AJ received research funding from Aplagon Ltd. The remaining authors declare that the research was conducted in the absence of any commercial or financial relationships that could be construed as a potential conflict of interest.

## Publisher’s Note

All claims expressed in this article are solely those of the authors and do not necessarily represent those of their affiliated organizations, or those of the publisher, the editors and the reviewers. Any product that may be evaluated in this article, or claim that may be made by its manufacturer, is not guaranteed or endorsed by the publisher.
